# CAIX在NSCLC中的表达及其与VEGF和Ki67表达的相关性

**DOI:** 10.3779/j.issn.1009-3419.2010.09.05

**Published:** 2010-09-20

**Authors:** 向飞 赵, 晓晴 刘, 万峰 郭, 晓兵 李

**Affiliations:** 1 100071 北京，北京军事医学科学院附属医院肺癌内科 Department of Lung Cancer, Affiliated Hospital, Academy of Military Medical Science, Beijing 100071, China; 2 100071 北京，北京军事医学科学院附属医院肺癌病理科 Department of Pathology, Affiliated Hospital, Academy of Military Medical Science, Beijing 100071, China

**Keywords:** 肺肿瘤, CAIX, VEGF, Ki67, 乏氧, Lung neoplasms, CAIX, VEGF, Ki67, Hypoxia

## Abstract

**背景与目的:**

已有的研究表明CAIX与肿瘤的乏氧代谢密切相关，细胞核增殖抗原(proliferating cell nuclear antigen, Ki67)被认为是能较可靠、全面地反映细胞群体增殖活性的客观指标；血管内皮生长因子(vascular endothelial growth factor, VEGF)与肿瘤血管生成呈正相关，本研究通过分析其与肺癌患者临床特点及与VEGF、Ki67表达的相关性，了解CAIX在非小细胞肺癌(non-small cell lung cancer, NSCLC)组织中表达的意义。

**方法:**

应用免疫组化SP方法检测76例NSCLC组织(33例鳞癌、43例腺癌)中CAIX、VEGF和Ki67表达及10例肺炎性假瘤中CAIX表达。

**结果:**

76例NSCLC组织中CAIX、VEGF和Ki67表达阳性率分别为46.1%、72.4%和39.5%；肺癌组织中CAIX表达明显高于炎性假瘤组(*P*＜0.001)；CAIX蛋白阳性表达率在鳞癌组和腺癌组表达分别为69.7%和27.9%(*P*=0.001)；在接受放疗的34例患者中，CAIX阳性组和阴性组放疗客观反应率分别为27.8%和62.5%(*P*=0.042)；CAIX蛋白表达与VEGF表达呈正相关(*r*=0.231, *P*=0.043)，但与Ki67表达无相关性(*r*=0.064, *P*=0.583)。

**结论:**

CAIX在NSCLC组织中表达较良性组织水平明显上升，并与VEGF表达相关；CAIX蛋白表达与放疗客观反应率相关，为乏氧增加NSCLC的放疗抗拒提供了新的证据。

## Abstract (en)

**Background and objective:**

It has been shown that carbonic anhydrase IX (CAIX) was closely related to tumor hypoxia. Proliferating cell nuclear antigen (Ki67) is considered to be an objective indicator which could reliable and comprehensive response to cell population proliferation; vascular endothelial growth factor (VEGF) was positively correlated with tumor angiogenesis. The aim of this study is to investigate the significance of the expression of CAIX in non-small cell lung cancer (NSCLC) tissues by analyzing the correlation between CAIX and clinical characteristics of patients with lung cancer and VEGF, Ki67 expressions.

**Methods:**

CAIX, VEGF and Ki67 protein were detected in 76 cases of NSCLC and 10 cases of lung inflammatory pseudotumor by immunohistochemistry.

**Results:**

CAIX was expressed in 45 of 64 cases of NSCLC and not expressed in benign lesion pulmonary tissues. VEGF was expressed in 55 of 76 cases and Ki67 was expressed in 30 of 76 cases. The expression of CAIX in squamous cell carcinoma (SC) was higher than that in adenocarcinoma (AC)(69.7% *vs* 27.9%, *P*=0.001). In 34 cases who received radiotherapy, the objective response rates of CAIX positive and negative groups were 27.8% and 62.5% respectively; the expression of CAIX was positively correlated with VEGF (*r*=0.231, *P*=0.043) and was not correlated with Ki67 (*r*=0.064, *P*=0.583).

**Conclusion:**

Compared with benign lesion pulmonary tissue, the expression of CAIX in NSCLC tissues is significantly increased and is positively correlated with VEGF. The expression of CAIX was also correlated with the objective response rate of radiotherapy, which provided new evidence about that hypoxia can increase NSCLC radiotherapy resistance.

碳酸酐酶IX(carbonic anhydrase IX, CAIX)是新发现的碳酸酐酶家族的异构体之一，与肿瘤的乏氧代谢密切相关。细胞核增殖抗原(proliferating cell nuclear antigen, Ki67)的表达出现于除G_0_期、G_1_期早期以外的细胞周期中，被认为是能较可靠、全面地反映细胞群体增殖活性的客观指标；血管内皮生长因子(vascular endothelial growth factor, VEGF)与肿瘤血管生成呈正相关。目前国内外关于CAIX在非小细胞肺癌(non-small cell lung cancer, NSCLC)中的表达及其与Ki67、VEGF表达的相关性研究较少，尤其是CAIX表达与肺癌治疗的相关性尚未见报道。本实验使用免疫组化的方法检测CAIX蛋白在NSCLC组织中的表达情况，分析其与放疗及增殖指标Ki67、血管增生指标VEGF的相关性，研究CAIX与NSCLC发病及治疗的相关性。

## 1 资料与方法

### 1.1 临床资料

选取我院肺癌内科1999年1月-2005年12月住院治疗并经病理证实的NSCLC 76例，男性53例，女性23例。年龄30岁-75岁，中位年龄59岁。其中腺癌43例，鳞癌33例；低分化43例，中分化16例，高分化17例；分期：Ⅰ期/Ⅱ期13例，Ⅲ期/Ⅳ期63例；接受放疗患者34例。患者均为初治患者。另取10例炎性假瘤标本作为阴性对照。

### 1.2 治疗方法

放疗方案为胸部病灶放疗剂量为60 Gy-72 Gy，颈、锁骨上淋巴结转移灶为60 Gy-70 Gy，脑转移灶为38 Gy-52 Gy，放疗方式为常规放疗与适形或调强放疗，分割方式有常规分割、超分割与低分割。部分放疗患者联合含铂类方案化疗。

### 1.3 主要试剂

兔抗人CAIX单抗、鼠抗人VEGF单抗、兔抗人Ki67单抗及PV-8000免疫组化试剂盒均购自北京中杉生物技术有限公司。CAIX工作浓度为1:50，稀释剂为PBS；VEGF、Ki67工作浓度为1:100，稀释剂为PBS。

### 1.4 实验方法

采用免疫组化SP法，标本切片为4 μm，经脱蜡、脱苯、水化后进行高压抗原修复，滴加抗体后置于4 ℃冰箱过夜。用已知染色阳性的胆囊上皮切片作为阳性对照，PBS代替一抗作阴性对照。

### 1.5 免疫组化结果判断

所有样本均经过两位病理医师进行评估。CAIX蛋白以细胞膜呈棕黄色为阳性标记(图 1A1和图 1B2)，VEGF以胞浆呈棕黄色颗粒为阳性标记(图 1A2和图 1B2)，Ki67以胞核呈棕黄色颗粒为阳性标记(图 1A3和图 1B3)，结果判断采用半定量积分法，在高倍镜下随机选择5个视野，每个视野计数200个细胞，共计1 000个，计算每张切片阳性细胞百分率，根据切片上阳性细胞所占百分比计分。阳性细胞≤5%为0分，6%-25%为1分，26%-50%为2分，51%-75%为3分，>75%为4分；显色度按细胞着色深浅计分：0分细胞无着色，1分浅黄色，2分棕黄色，3分棕褐色。两者积分相乘，0分-4分为阴性，5分-12分为阳性。

**1:**
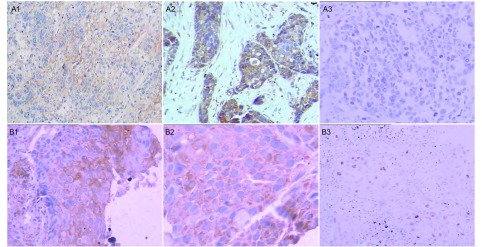
CAIX、VEGF和Ki67在肺腺癌及肺鳞癌组织中的表达。A：腺癌；B：鳞癌；1：CAIX(+)；2：VEGF(+)；3：Ki67(+)。A1：SP，×200；A2：SP，×400；A3：SP，×200；B1：SP，×200；B2：SP，×400；B3：SP，×200。 The expression of CAIX, VEGF and Ki67 in NSCLC. A: adenocarcinoma; B: squamous cell carcinoma; 1: CAIX (+); 2: VEGF (+); 3: Ki67 (+). A1: SP, × 200; A2: SP, ×400; A3: SP, ×200; B1: SP, ×200; B2: SP, ×400; B3: SP, ×200.

### 1.6 疗效评价标准

按照世界卫生组织(WHO)抗肿瘤治疗的客观疗效标准进行评价。完全缓解(complete response, CR)：完全消失，无新病灶，无疾病相关症状，至少持续4周；部分缓解(partial response, PR)：与基线相比，病灶两个最大垂直横径乘积总和减少＞50%，且持续4周；稳定(stable disease, SD)：与基线相比，未达CR、PR，无疾病进展，无新病灶形成；进展(progressive disease, PD)：与基线相比，病灶两个最大垂直横径乘积增大＞25%，或任何可评估的病灶出现恶化或发现新病灶。以疗效CR+PR为有效组，疗效SD+PD为无效组。

### 1.7 统计学分析

采用SPSS 11.5软件对数据进行统计分析，计数资料采用*χ*^2^检验，均数比较采用*t*检验，等级相关用*Spearman*分析，以*P*＜0.05为有统计学差异。

## 2 结果

### 2.1 NSCLC及炎性假瘤组织中CAIX蛋白的表达

76例NSCLC组织中，35例CAIX表达呈阳性，阳性率为46.1%，而在良性病变炎性假瘤组中无表达，两者差异有统计学意义(*P*＜0.001)。

### 2.2 CAIX蛋白在NSCLC中的表达

CAIX蛋白表达在鳞癌组的阳性率为23/33(69.7%)，明显高于腺癌组12/43 (27.9%)，差异有统计学意义(*P*=0.001)；CAIX蛋白表达在不同分化程度之间差异具有统计学意义(*P*=0.005)；但CAIX蛋白表达在性别、年龄、TNM分期、淋巴结转移、远处转移、吸烟、体重下降各组间表达差异无统计学意义(表 1)。

**1:** CAIX与NSCLC患者临床病理特征相关性 Relationship between expression of CAIX with clinicopathologic characteristics in NSCLC

Characteristics	*n*	Expression of CAIX		*χ*^2^	*P*
		(+)	%		
Age (year)				0.117	0.732
≥70	15	8	53.3		
＜70	61	27	44.3		
Gender				0.002	0.963
Male	53	25	47.2		
Female	23	10	43.5		
Histological type				11.496	0.001
Squamous cell carcinoma	33	23	69.7		
Adenocarcinoma	43	12	27.9		
Differentiation				10.609	0.005
High	17	5	29.4		
Moderate	16	13	81.3		
Poor	43	17	39.5		
Clinical stage				0.098	0.754
Ⅰ+Ⅱ	13	7	53.8		
Ⅲ+Ⅳ	63	28	44.4		
Metastasis				2.791	0.095
Positive	35	12	34.3		
Negative	41	23	56.1		
KPS				0.441	0.506
＜80	19	7	36.8		
≥80	57	28	49.1		
Smoking				3.390	0.066
Yes	38	22	57.9		
No	38	13	34.2		
Weight lose				0.008	0.930
Yes	21	9	42.9		
No	55	26	47.3		
CAIX: carbonic anhydrase IX.					

### 2.3 CAIX蛋白表达与放疗疗效的相关性

CAIX阳性组18例，客观反应率(objective response rate, ORR) (CR+PR)为27.8%，CAIX阴性组16例，阳性例数13例，阳性率62.5%，两组相比差异有统计学意义(*P*=0.042) (表 2)。

**2:** CAIX表达与放疗疗效客观反应率的相关性 Relationship between expression of CAIX with ORR of radiotherapy in NSCLC

	Total (*n*)	CAIX		*r*	*P*
		CR+PR (*n*)	%		
CAIX (+)	18	5	27.8	4.142	0.042
CAIX (-)	16	10	62.5		
CR: complete response; PR: partial response.					

### 2.4 NSCLC中VEGF和Ki67的表达与临床病理因素的关系

76例NSCLC组织中，55例VEGF表达呈阳性，阳性率为72.4%，与是否存在体重下降具有相关性(*P*=0.016)，在性别、年龄、组织学类型、分化程度、TNM分期、淋巴结转移、远处转移、吸烟、KPS评分方面，各组间表达差异无统计学意义。在76例NSCLC组织中有32例Ki67表达阳性，阳性率为42.1%，在性别、年龄、组织学类型、分化程度、TNM分期、淋巴结转移、远处转移、吸烟、KPS评分、体重下降方面，各组间表达差异均无统计学意义。

### 2.5 NSCLC组织中CAIX蛋白表达与VEGF、Ki67表达的相关性

76例NSCLC组织中，55例患者VEGF表达阳性，其中CAIX表达阳性者为29例(52.7%)，在VEGF表达阴性的21例患者中，CAIX表达阳性者为16例，二者表达呈正相关(*r*=0.231, *P*=0.043)；CAIX和Ki67共同表达阳性者为15例，在Ki67表达阴性的46例中，CAIX表达阳性者为20例，二者表达无相关性(*r*=0.064, *P*=0.583)(表 3，表 4)。

**3:** CAIX蛋白表达与VEGF表达的相关性 Relationship between expression of CAIX with VEGF in NSCLC

	CAIX (+)	CAIX (-)	Total
VEGF (+)	29	26	55
VEGF (-)	6	15	21
Total	35	41	76
*r*=0.231, *P*=0.043.			

**4:** CAIX蛋白表达与Ki67表达的相关性 Relationship between expression of CAIX with Ki67 in NSCLC

	CAIX (+)	CAIX (-)	Total
Ki67 (+)	15	15	30
Ki67 (-)	20	26	46
Total	35	41	76
*r*=0.064, *P*=0.583.			

## 3 讨论

CAIX最早被称为MN蛋白，是1993年由Pastorekova在与人乳腺癌细胞联合培养的人宫颈癌的HeLa细胞系中发现的^[1]^。CAIX蛋白在正常人上消化道及消化道相关器官如胰腺、胆囊、肝脏中大量表达，正常人心脏、肺、肾、前列腺、外周血、脑、胎盘及肌肉组织中不表达CAIX^[2]^。CAIX在NSCLC中的表达情况报道不一，现有的文献^[3-5]^报道，CAIX蛋白在NSCLC组织中的表达阳性率为36.4%-81%，我们的研究使用免疫组化的方法检测CAIX在NSCLC中的表达，结果显示在NSCLC患者中CAIX表达阳性率达46.1%，10例良性病变无一例存在CAIX表达，提示CAIX有作为临床肺部病变鉴别良恶性指标的潜力，鳞癌患者阳性率明显高于腺癌患者，这与Giatromanolaki研究^[5]^的结果是一致的，在不同的分化程度的患者中，CAIX表达阳性率也有差异，表明CAIX与NSCLC的发生或发展存在相关性。CAIX的表达与肿瘤的乏氧代谢密切相关，在实体肿瘤中，多数肿瘤细胞生长迅速，血管的生长速度相对滞后，以致内部肿瘤细胞供血不足，导致肿瘤细胞乏氧，古模发^[6]^使用乏氧组织显像剂^99m^Tc-HL91对照检测了41例肺癌患者和33例非肺癌患者，发现对照组33例均为无明显乏氧；肺癌组则无明显乏氧0例，低度乏氧18例，高度乏氧23例，各组分布有明显差异。Le^[7]^在早期NSCLC患者术中直接用电极测量癌组织的氧浓度，分析了其与肺癌组织CAIX蛋白表达的关系，发现二者存在负相关，认为在NSCLC中CAIX表达与乏氧相关，可以作为乏氧代谢的观测指标。在我们的研究中，CAIX表达与NSCLC的相关性间接提示了NSCLC组织中存在乏氧，鳞癌患者肿瘤组织中乏氧存在的比例可能高于腺癌患者。

乏氧是导致实体肿瘤治疗抵抗的重要原因，我们进一步研究了CAIX表达与NSCLC放疗疗效的相关性。近来的研究^[8]^表明NSCLC患者CAIX表达与生存期相关，但尚无研究分析其与治疗疗效的关系，我们的研究发现放疗疗效的客观反应率在CAIX表达阳性组和阴性组之间的差异有统计学意义，国外研究^[10]^发现在头颈部鳞癌中CAIX表达与放疗疗效具有相关性，本研究显示CAIX阴性组的客观反应率明显高于CAIX阳性组，提示CAIX表达阳性对放疗疗效可能存在一定的预测作用，由于CAIX表达与乏氧代谢的密切相关性，本研究为乏氧增加NSCLC的放疗抵抗提供了新的证据。

乏氧可使肿瘤细胞的一些基因和蛋白的表达发生改变，包括CAIX、VEGF在内的多个基因表达的上调^[11]^。在Kim的研究^[8]^中，使用RT-PCR技术检测了74例早期NSCLC患者的术后切除标本中CAIX和V EG F的表达，发现CAIX mRNA的表达与VEGF mRNA呈正相关(*P*=0.002)，本研究发现CAIX蛋白表达与VEGF蛋白表达呈正相关，与Kim的研究结论一致，提示在NSCLC中VEGF的表达可能与乏氧相关。CAIX与VEGF表达的正相关关系可能是由于它们结构的侧链均存在一个乏氧反应元件(hypoxia-responsive element, HRE)，可以在乏氧的条件下被激活，从而使表达共同上调^[12]^，而不是线性的激活与被激活的关系。多个研究^[13, 14]^表明在肾癌、乳腺癌中CAIX的表达与Ki67表达相关，但我们的研究却发现在NSCLC患者中CAIX表达与Ki67表达差异无统计学意义，在Kim的另外一个研究^[15]^中也发现在75例NSCLC患者中CAIX表达与Ki67表达差异无统计学意义，但对CAIX高表达的30例患者分析发现，CAIX染色阳性区域较阴性区域有更高比例的Ki67表达，提示CAIX表达有助于肿瘤细胞的增殖，本研究因CAIX(+++)表达例数仅10例，故未进行此项观察。

本研究的结果表明：CAIX在NSCLC组织中表达较良性组织水平明显上升，并与VEGF表达相关；CAIX蛋白表达与客观反应率相关，为乏氧增加NSCLC的放疗抵抗提供了新的证据。

## References

[1] Barnea G, Silvennoinen O, Shaanan B (1993). Identification of a calbonic anhydrase-like domain in the extracellular region of RPTP gamma defines a new subfamily of receptor tyrosine phosphatases. Mol Cell Biol.

[b2] 2Pastorekova S, Pastorek J. Cancer-related carbonicanhydrase isozymes and their inhibition[A]. Supuran CT. Carbonic anhydrase. Its Inhibitors and activators[M]. RocaRaton, FL: CRC Press, 2004. 255-281.

[3] Swinson DE, Cox G, O'Byrne KJ (2004). Coexpression of epidermal growth factor receptor with related factors is associated with a poor prognosis in non-small-cell lung cancer. Br J Cancer.

[4] Simi L, Venturini G, Malentacchi F (2006). Quantitative analysis of carbonic anhydrase IX mRNA in human non-small cell lung cancer. Lung Cancer.

[5] Giatromanolaki A, Koukourakis MI, Sivridis E (2001). Expression of hypoxiainducible carbonic anhydrase-9 relates to angiogenic pathways and independently to poor outcome in non-small cell lung cancer. Cancer Res.

[6] Gu MF, Li JJ, Gao JM (2007). ^99m^Tc-HL91 hypoxic scintigr aphy for lung cancer. J SUN Yat-sen Univ (Med Sci).

[7] Le QT, Chen E, Salim A (2006). An evaluation of tumor oxygenation and gene expression in patients with early stage non-small cell lung cancers. Clin Cancer Res.

[8] Kim SJ, Rabbani ZN, Dewhirst MW (2005). Expression of HIF-1 alpha, CAIX, VEGF, and MMP-9 in surgically resected non-small cell lung cancer. Lung Cancer.

[9] Raghunand N, Mahoney BP, Gillies RJ (2003). Tumor acidity, ion trapping and chemotherapeutics. Ⅱ. pH-dependent partition coefficients predict importance of ion trapping on pharmacokinetics of weakly basic chemotherapeutic agents. Biochem Pharmacol.

[10] Koukourakis MI, Bentzen SM, Giatromanolaki A (2006). Endogenous markers of two separate hypoxia response pathways (hypoxia inducible factor 2 alpha and carbonic anhydrase 9) are associated with radiotherapy failure in head and neck cancer patients recruited in the CHART randomized trial. J Clin Oncol.

[11] Said HM, Hagemann C, Staab A (2007). Expression patterns of the hypoxiarelated genes osteopontin, CA9, erythropoietin, VEGF and HIF-1 alpha in human glioma *in vitro* and *in vivo*. Radiother Oncol.

[12] Kaluzova M, Pastorekova S, Svastova E (2001). Characterization of the MN/CA 9 promoter proximal region: a role for specificity protein (SP) and activator protein 1 (AP1) factors. Biochem J.

[13] Generali D, Fox SB, Berruti A (2006). Role of carbonic anhydrase IX expression in prediction of the efficacy and outcome of primary epirubicin/tamoxifen therapy for breast cancer. Endocr Relat Cancer.

[14] Bui MH, Visapaa H, Seligson D (2004). Prognostic value of carbonic anhydrase IX and KI67 as predictors of survival for renal clear cell carcinoma. J Urol.

[15] Kim SJ, Rabbani ZN, Vollmer RT (2004). Carbonic anhydrase IX in early-stage non-small cell lung cancer. Clin Cancer Res.

